# Central Diabetes Insipidus in a Patient With Lymphoma: A Case Report

**DOI:** 10.7759/cureus.41500

**Published:** 2023-07-07

**Authors:** Epameinondas Koumpis, Lydia Kyriazopoulou, Stelios Tigas, Eleni Kapsali, Eleftheria Hatzimichael

**Affiliations:** 1 Department of Hematology, Faculty of Medicine, School of Health Sciences, University of Ioannina, Ioannina, GRC; 2 Department of Endocrinology, Faculty of Medicine, School of Health Sciences, University of Ioannina, Ioannina, GRC

**Keywords:** neurolymphomatosis, hypernatremia, central diabetes insipidus, dlbcl, lymphoma

## Abstract

Primary central nervous system (CNS) lymphoma or systemic non-Hodgkin lymphoma that infiltrates the CNS can cause central diabetes insipidus (CDI). Polyuria and polydipsia should raise the suspicion of CDI development in patients with lymphoma that infiltrates the CNS. CDI is effectively treated with desmopressin. However, careful monitoring of the patient's serum sodium, fluid intake, urine output, and weight is necessary because patients receiving desmopressin may develop hyponatremia, so they should be alert to recognize this side effect promptly. Moreover, CDI due to lymphoma can occasionally be reversible. Therefore, the dosage of desmopressin should be adapted during or after the treatment of lymphoma.

## Introduction

Primary central nervous system (CNS) lymphoma or systemic non-Hodgkin lymphoma that infiltrates the CNS can involve the hypothalamus-pituitary axis and cause central diabetes insipidus (CDI) [1,2]. Clinical findings of CDI include polyuria, polydipsia, and nocturia, as well as hypernatremia in cases of dehydration [3]. Clinical hematologists should recognize this condition since hypernatremia is an independent risk factor for mortality, while both hypernatremia and polyuria are manageable [4].

## Case presentation

Chief complaints and disease diagnosis

A 60-year-old female was referred to our department due to neurolymphomatosis of the left sciatic nerve. For the last four months, the patient suffered from sciatica, and a nerve biopsy finally revealed diffuse large B-cell lymphoma (DLBCL) of the activated B-cell (ABC) subtype. Immunohistochemical (IHC) analysis of the biopsy showed a markedly elevated proliferation index (Ki67), and the immunophenotype of neoplastic cells was as follows: CD20+, PAX5+, BCL6+, MUM1/IRF4+, CD5-, CD10-, and pankeratin negative. The stage by the Ann Arbor Staging System was IV, the International Prognostic Index was 5, and the bone marrow karyotype was 48, XX,+3,+12/46XX.

Physical examination at the admission and past medical history

On physical examination, she had dysmetria and muscle weakness in the left upper and lower limp. Past medical histories included primary hypothyroidism, dyslipidemia, and gastritis. Her medication included methylprednisolone (4 mg per day) due to possible bronchiolitis obliterans organizing pneumonia; levothyroxine sodium (50 μg once daily [od]), atorvastatin (40 mg od), acetylsalicylic acid (100 mg od), and pantoprazole (40 mg od). A few weeks before her admission, she was treated with pregabalin for sciatica.

Laboratory and imaging workup

A computed tomography (CT) scan of the brain revealed periventricular enhancement and edema. Biochemical, microbiological, and immunological laboratory tests did not reveal any abnormalities except for reduced levels of thyroid-stimulating hormone (TSH) and increased levels of serum lactate dehydrogenase (LDH) (Table 1). A lumbar puncture was conducted, and microscopy of the cerebrospinal fluid showed 57 leucocytes (98% lymphocytes), while cytology was suspicious of lymphoma cell infiltration. A polymerase chain reaction of 14 pathogens in the cerebrospinal fluid was also performed (including viruses such as Cytomegalovirus, Human Herpes Virus 6, and Varicella Zoster Virus), and the result was negative.

**Table 1:** Initial laboratory findings at admission. Microbiological and immunological tests were negative. ALP: alkaline phosphatase, ALT: alanine transaminase, AST: aspartate aminotransferase, DBIL: direct bilirubin, LDH: lactate dehydrogenase, TBIL: total bilirubin, TSH: thyroid-stimulating hormone, γGT: gamma-glutamyl transferase

Laboratory findings	Values	Normal range
Hemoglobin (g/dL)	15.9	12-16.5
White blood cells(/μL)	13,500	4,500-11,000
Platelets (/μL)	256,000	150,000-450,000
Creatinine (mg/dL)	0.66	0.6-1.2
Urea (mg/dL)	35	11-54
AST/ALT (IU/L)	29/28	10-35/10-35
γGT/ALP (IU/L)	31/61	6-32/30-125
TBIL/DBIL (mg/dL)	0.9/0.12	0.1-1/0.01-0.2
LDH (U/L)	291	115-230
Potassium (mmol/L)	4.51	3.5-5.3
Sodium (mmol/L)	138	136-146
Calcium (mg/dL)	8.6	8.2-10.5
Total protein/Albumin (g/dL)	6.0/3.9	6-8.4/3.4-5
Direct Coombs	negative	
TSH (μIU/mL)	0.05	0.38-5.33

Management of lymphoma and complication of hypernatremia

Her management plan was to receive chemoimmunotherapy (R-CHOP) alternating with high-dose methotrexate and high-dose aracytin. She started with high-dose methotrexate and high-dose cytarabine. On day three of treatment, she developed hypernatremia, which was considered to be related to the administration of saline solutions, including sodium bicarbonate, and was corrected appropriately. However, a few days later, hypernatremia recurred, and polyurea was added to the patient’s symptoms (11L/24 hours). Plasma osmolarity (Posm) was 300 mosmol, and urine osmolarity (Uosm) was 173 mosmol, consistent with the diagnosis of diabetes insipidus. Following the administration of 1 mcg of desmopressin (DDAVP), the Posm was 296 mosmol, and the Uosm was 520 mosmol, supporting the diagnosis of CDI [3,5]. Luteinizing hormone (LH), follicle-stimulating hormone (FSH), TSH, adrenocorticotropic hormone (ACTH), and growth hormone (GH) were in low serum concentrations, supporting the diagnosis of panhypopituitarism (Table 2).

**Table 2:** Laboratory findings suggest panhypopituitarism. ACTH: adrenocorticotropic hormone, FSH: follicle-stimulating hormone, GH: growth hormone, LH: luteinizing hormone, TSH: thyroid-stimulating hormone

Laboratory findings	Values	Normal range
LH (IU/L)	1.0	10.9-58.6
FSH (IU/L)	4.9	16.7-113.6
TSH (μIU/mL)	0.25	0.38-5.33
ACTH (pg/mL)	9.0	0-46
GH (ng/mL)	0.54	0.01-3.61

Management of CDI

**Figure 1:**
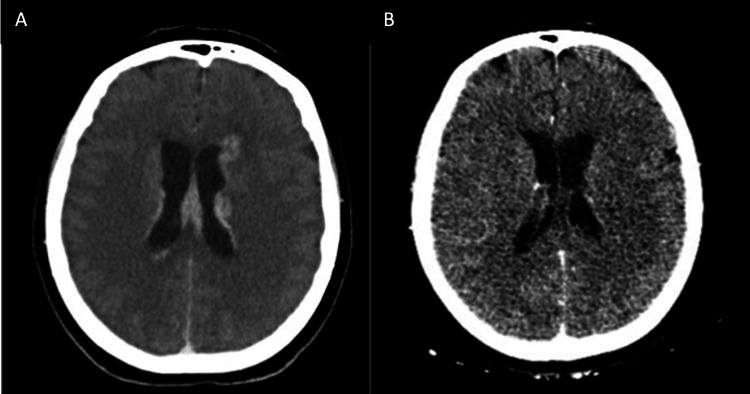
The computed tomography scan of the brain revealed periventricular enhancement and edema. B. Five months later, remission of periventricular enhancement and edema were noticed.

The patient was treated with DDAVP Melt 60 micrograms of oral lyophilizate twice daily, which was reduced to a dose of 60 micrograms once daily due to rapid correction of hypernatremia. DDAVP Melt was administered in the evening due to nocturia. Magnetic resonance imaging (MRI) of the brain and pituitary gland was available after the first cycle of therapy. The MRI revealed subependymal contrast enhancement of the frontal and occipital horns of the lateral ventricles as well as the anterior part of the third ventricle. The patient responded to chemoimmunotherapy, and a few months later, she gradually discontinued DDAVP Melt while both neurologic symptoms and imaging studies improved (Figure 1).

## Discussion

CDI is characterized by decreased release of ADH, resulting in polyuria and nocturia. Lack of ADH can be caused by various mechanisms, including decreased secretion from the hypothalamus, decreased storage of ADH in the posterior pituitary, or decreased ADH transport to the posterior pituitary gland [3,6].

**Figure 2:**
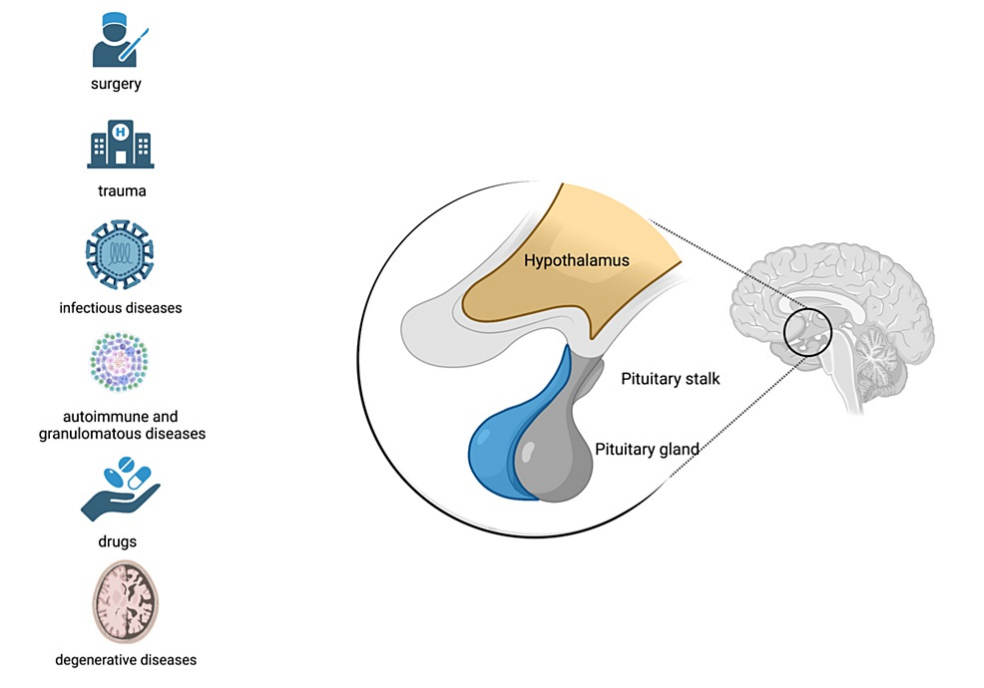
Causes of CDI and the hypothalamus-pituitary axis Primary central nervous system lymphoma or systematic non-Hodgkin lymphoma that infiltrates the hypothalamus-pituitary axis can cause central diabetes insipidus (CDI). Infiltration may involve the paraventricular nucleus and supraoptic nucleus in the hypothalamus, pituitary stalk, or pituitary gland. Other causes of CDI include surgery, trauma, other neoplasia, infectious diseases (meningitis, encephalitis), autoimmune and granulomatous diseases (e.g., sarcoidosis), drugs, and degenerative diseases.

In our case, the differential diagnosis of CDI etiology was wide, including a variety of causes apart from lymphoma infiltration of the CNS. An idiosyncratic effect of high-dose methotrexate administration could not be excluded as the cause of the observed CDI [7]. Diabetes insipidus due to herpes encephalitis in a patient with DLBCL has also been reported [8]. However, in our case, the polymerase chain reaction of many pathogens (including Varicella Zoster Virus) in the cerebrospinal fluid was negative. Our patient had pulmonary lesions, while tuberculosis had been excluded as the cause. Angiotensin-converting enzyme (ACE) levels, as well as serum and urine calcium, were within normal limits; however, CDI due to sarcoidosis and neurosarcoidosis could not be excluded [9,10] (Figure 2).

Primary or secondary CNS tumors can infiltrate the hypothalamus or pituitary gland and cause CDI. In many patients, polyuria is the first symptom [2]. Among the cancers that infiltrate the pituitary, lymphomas represent only 0.5% [11]. Primary CNS lymphoma and secondary infiltrative lymphoma can infiltrate the hypothalamus and induce CDI with panhypopituitarism [9,12]. Moreover, lymphoma can infiltrate the pituitary stalk, depicted as thickening in imaging studies and causing hypopituitarism along with CDI [13]. Infiltration of the pituitary stalk by lymphomas may also have a specific V-shaped pattern in magnetic resonance imaging (MRI) [14]. A case of infiltration of the pituitary stalk infiltration of DLBCL leading to hypopituitarism and CDI, along with a radiologically smaller size of the hypophysis, has also been reported [15]. In addition, imaging of a patient with lymphoma and CDI revealed a hypophyseal mass that was quite reversible after the treatment [16]. Except for primary CNS lymphoma and DLBCL, Burkitt lymphomas and follicular lymphomas that infiltrate the CNS may cause CDI [17,18].

The correction of hypernatremia is critical as it is associated with increased mortality, especially when hospital-acquired [4,19]. Management of CDI includes administration of DDAVP Melt, correction of dehydration when necessary, as well as careful monitoring of serum sodium, fluid intake, urine output, and the patient’s weight. The most appropriate treatment is DDAVP Melt 60-120 micrograms twice or three times a day, with 60 micrograms per day being adequate quite often. Patients with CDI usually drink a lot of coffee and soda. When desmopressin is administered, patients should be advised to limit their consumption of drinks due to the accumulative risk of hyponatremia. Moreover, they should be educated to promptly recognize symptoms of hyponatremia [3,6,20]. Finally, infiltrative diseases, such as lymphomas, can cause partially reversible central diabetes insipidus. In that case, the administration of desmopressin should be adapted or stopped with careful monitoring of serum sodium levels [16]. Clinical practice points are summarized in Table 3.

**Table 3:** Clinical practice points of the case report

Clinical practice points
In primary central nervous system (CNS) lymphoma or systemic non-Hodgkin lymphoma that infiltrates the CNS, polyurea, and polydipsia should raise the suspicion of central diabetes insipidus (CDI) development.
CDI is effectively treated with desmopressin. However, careful monitoring of serum sodium, fluid intake, urine output, and the patient’s weight is essential.
CDI can occasionally be partially reversible. Therefore, the dosage of desmopressin should be adapted during or after the treatment of lymphoma.

## Conclusions

In patients with lymphoma, polyuria, and hypernatremia should be recognized as possible manifestations of CDI. Reaching a diagnosis of CDI is essential and critical since it is a condition associated with mortality that can be managed pharmaceutically. Desmopressin is the most appropriate treatment; however, patients receiving it should be informed of manifestations of hyponatremia to recognize them promptly. The treatment of lymphoma could sometimes reverse the CDI; therefore, physicians should be alerted to adapt the dosage. In our case, the patient responded to chemoimmunotherapy, and a few months later, she gradually discontinued desmopressin. This discontinuation could be considered indicative of a complete response.
